# Dental public health education in Egypt: a cross-sectional survey

**DOI:** 10.1186/s12909-023-04888-9

**Published:** 2023-11-25

**Authors:** Haya Gouda, Jorma I. Virtanen, Maha El Tantawi

**Affiliations:** 1 Faculty of Dentistry, Alexandria University, Alexandria, Egypt; 2 Faculty of Medicine, University of Bergen, Bergen, Norway; 3 Institute of Dentistry, University of Turku, Turku, Finland

**Keywords:** Dental Public Health, Education, Assessment, Teaching, Lectures, Academics

## Abstract

**Background and aim:**

Dental Public Health (DPH) education prepares future workforce to promote positive oral health behaviors, prevent oral diseases, and monitor disease distribution and trends taking into considerations best practices, needs and available resources. Scarce information is available about dental education in African countries and Egypt has the greatest number of dental schools in Africa. This study assessed the undergraduate DPH education in Egyptian universities including topics taught, methods of teaching, assessment, and the academics’ specialties.

**Methods:**

A survey targeted 43 Egyptian universities with Bachelor of Dentistry (BDS) programs identified on the website of the Supreme Council of Egyptian Universities in 2022. Thirty-six deans could be reached by post and/ or email. The survey appraised the school profile and capacity, and methods of teaching and assessment in DPH courses in undergraduate dental programs. The survey also inquired who taught DPH courses and what was covered in the courses. Descriptive statistics were displayed.

**Results:**

We received 21 (58.3%) responses from 36 deans/ senior officials. Of the universities, 52.4% were private and 47.6% were public. Most participants reported that DPH courses in BDS programs were taught by Pediatric Dentistry academics (71.4%) and DPH academics (57.1%) in 3rd, 4th and 5th years of the 5-year BDS programs. Teaching DPH consisted of face-to-face lectures (100%) and seminars (95.2%) and assessment included written exams with close ended questions (95.2%) and open-ended questions (71.4%). Twenty schools reported teaching the definition of DPH, definition of oral health, and determinants of oral diseases. Nine schools addressed the planning of oral health services and five schools taught about remuneration and payment systems.

**Conclusion:**

Teaching and assessment of DPH in Egyptian dental schools use traditional methods with limited active engagement of the students. Variations among the schools exist in the DPH topics covered and most instructors were not primarily specialized in DPH. Development of dental/ oral health services calls for more emphasis on DPH education in the curriculum in Egypt.

**Supplementary Information:**

The online version contains supplementary material available at 10.1186/s12909-023-04888-9.

## Introduction

Dental education aims to facilitate and assess the learning of dental sciences, allowing learners to gain the cognitive and psychomotor skills needed to practice dentistry [1]. Dental Public Health (DPH) is one of the dental specialties recognized in North America [2] and Europe [3]. It trains learners to educate the community about oral health and diseases, promote healthy behaviors, assess the distribution and determinants of oral diseases, evaluate and build efficient healthcare services and encourage and empower people to make decisions about their oral health. A robust DPH curriculum would positively influence the oral health of the community.

Dentistry and oral healthcare are at a turning point after the resolution of the World Health Assembly in 2021 [4] and the impending adoption of the global oral health action plan [5]. The action plan calls for the incorporation of oral health within the non-communicable diseases’ agenda, the inclusion of oral healthcare under universal health coverage, making essential oral healthcare services available as part of primary healthcare services and developing innovative oral healthcare workforce models. The global oral health action plan emphasizes the importance of adopting a public health approach to oral health [6]. This paradigm shift requires the training of dental graduates in the skills and techniques of DPH. The DPH competence and skills of dental graduates are, in turn, dependent on the DPH education they receive.

At a time of globalization and increased mobility of professionals [7], it is important to ensure that educational institutions in different countries follow international educational standards for teaching and assessment so that the quality of graduates is comparable across regions and systems. Previous research [8–11] showed variation among countries in DPH education including topics covered, methods of teaching and assessment, academics responsible for teaching the subject matter and allocated time. However, available information is almost exclusively from Europe and North America with scarce data available about DPH education in other locations. On the other hand, evidence from the recent Global Oral Health Status Report [12] showed that the greatest number of persons with oral diseases (untreated caries in primary and permanent teeth, severe periodontitis and tooth loss) exists in African countries. These countries also have the lowest expenditure on oral healthcare and a high percentage of out-of-pocket spending for oral healthcare with the lowest dentist to population ratios globally. The oral health problems of African countries clearly show a great need for a DPH solution that can only be achieved if dental graduates receive adequate DPH training during their BDS studies. However, no data are available about the type or extent of DPH training in undergraduate dental programs in Africa.

A recent study reported that 22 countries in the World Health Organization (WHO) African region had 61 dental schools [13]. Egypt, which is included in the WHO Eastern Mediterranean region, has 43 universities with undergraduate dental programs [14] indicating the large size of the dental academic sector in the country. In addition, large numbers of international students from neighboring countries finish their university studies and higher degrees in different programs in Egypt. Thus, the structure and content of dental curricula in Egyptian universities affect its population, which is one of the largest globally and in the region [15] as well as the populations of neighboring countries in Africa and the Middle East. Assessing DPH education in Egypt may help shed light on DPH education in North African countries and Africa in general.

This study assessed the education of DPH in Egyptian universities regarding the topics covered, methods of teaching and assessment, academics responsible for teaching DPH and the relative time allocated for various topics.

## Materials and methods

### Design, setting and ethical considerations

This cross-sectional study targeted 43 Egyptian universities with Bachelor of Dental Surgery (BDS) programs. The study was approved by the Research Ethics Committee, Faculty of Dentistry Alexandria University, Egypt (#0546 − 11/2022). Data was collected using an online questionnaire from September 2022 to January 2023. Participants provided their informed consent at the beginning of the electronic questionnaire by checking a box. The study was conducted in accordance with the Helsinki declaration.

### Participants

We utilized the website of the Supreme Council of Egyptian Universities [16–18] that listed all universities in Egypt. Each university website was checked to determine if it had a dental program. All schools with ministerial approvals for BDS programs were targeted, and no sample was used. Thus, to be included in the study, a dental school had to be in Egypt, operating during the study period, based on a ministerial decree, and part of a university listed on the website of the Supreme Council of Egyptian Universities. There were no exclusion criteria. In Egypt, BDS programs are five year long, specialty-based and followed by a 6th year of internship where integrated care is coordinated among all dental specialties to create a treatment plan addressing the patients’ condition. Some BDS programs in Egypt are year-based and others are semester-based using credit hour systems depending on the regulations of the universities offering the programs. Most BDS programs are managed by the vice dean for education and students’ affairs who coordinates with academic departments to cover topics in courses based on approved bylaws. Thus, the content, teaching responsibility and assessment methods are mostly predetermined by the bylaws, supervised by vice deans, and implemented by assigned academics in various departments. The first public dental school was established in 1925 in Cairo University. In the late 1990s, private universities received approvals to open BDS programs, and the number of private dental schools has increased ever since with tuition fees much higher than the state-supported public schools. In general, private dental schools and those that are newly opened tend to have BDS programs with no postgraduate programs and therefore, limited research activities.

The contact information, including the postal address of the school and the email of the dean, was obtained from the institutional websites of the universities. Missing information was sought on the universities’ Facebook pages. The deans were invited to reply to the survey and asked to invite other academics to respond to the survey as needed including vice deans for education and students’ affairs, heads of departments, program directors or senior academics teaching DPH.

### Study questionnaire

The questionnaire was developed in English based on a previous European study [9] and modified to suit the educational system in Egypt. The questionnaire was divided into three sections (Appendix 1). The first section included four questions assessing the profile of each dental school including its name, affiliation, number of dental students and mission. The second section asked about the department or division responsible for teaching DPH, number and specialty of academics teaching DPH, program level where DPH is taught, methods of teaching, and assessment using eight questions. The last section included four questions about the basis of developing the DPH undergraduate course/s, the topics covered in the curriculum and the duration for each, further comments and the title or role of the respondent. We culturally adapted the original questionnaire by removing options in some questions that did not fit the Egyptian context such as a question about when DPH is taught (option of 6th year) and a question about who teaches DPH (option of experts in Sociology or Epidemiology). Content validity was assessed by calculating the content validity index (CVI) [19] based on feedback from six DPH academics at Alexandria University. The CVI was 0.87 indicating good content validity. The questionnaire was finally uploaded to Google Forms.

### Data collection

An email was sent to the deans of all identified dental schools including a link to access the questionnaire. The email message explained the study purpose, mentioned the name of the PI and her contact information, and invited the deans to provide the required information by filling in the Google Form. In addition to reaching out to the deans by email and using an electronic questionnaire, the invitation to participate was sent by postal services to the street address of each dental school. The same message included in the email was printed and edited so that the link was replaced by a QR code that participants could scan to directly access the questionnaire to avoid errors in copying the link. A few dental schools were contacted through their academics on WhatsApp because no email or street addresses for the dental school could be identified. The survey was followed by two reminders, one month apart, to improve the response rate. A customized message was addressed to the deans of non-responding schools in the reminders to further increase response rates.

Two weeks after the last reminder, data were downloaded and cleaned for analysis. Descriptive statistics were calculated as medians and interquartile ranges for quantitative variables and numbers and frequencies for categorical variables. Data about non-responding schools/ programs were collected to identify potential reasons for non-response including recent establishment of BDS programs that have not yet started teaching DPH.

## Results

Out of the 43 eligible dental schools, contact information of two schools was not available. For the remaining 41 schools, only postal addresses were available for three, but the letters were returned undelivered. For two schools, only deans’ email addresses were available, but the emails bounced back. Thus, deans of 36 dental schools could be reached and 21 (58.3%) responded (Appendix 2). The remaining 15 nonresponding dental schools included 8 (53.3%) that were established within the last 4 years and have, therefore, not graduated any students and may have not started teaching DPH yet.

Table 1 shows that most responding schools were private and with undergraduate programs. Among schools with graduates (n = 19, 90.5%), the median number of graduates per year was 350 with a total of 7,562 graduates all over Egypt in the academic year 2021 [20].

**Table 1:** The profile of the Egyptian dental schools participating in the survey

Factor	N (%)
Type	
Affiliated with religious bodies (Al-Azhar)	2 (9.5)
Private	11 (52.4)
Public	8 (38.1)
Mission ^¶^	
Teaching	20 (95.2)
Undergraduate	19 (90.5)
Postgraduate	17 (81.0)
Research	14 (66.7)
Number of graduates/ year: median (min, max)	350 (65, 650)

^¶^ Percentages do not add up to 100 because multiple responses were allowed

Table 2 shows that teaching DPH in BDS programs is mostly assigned to various divisions within academic departments. The departments were Pediatric Dentistry (n = 5) without or with Orthodontics (n = 1), Oral Health (n = 1), or Preventive Dentistry (n = 2). The median number of academics teaching DPH was 3 per university. Most dental schools indicated that DPH was taught by Pediatric Dentistry academics and DPH academics.

**Table 2:** Administrative units and academics teaching DPH in the Egyptian dental schools participating in the survey

Factor	N (%)
Administrative unit teaching DPH	
Department	2 (9.5)
Division within department	16 (76.2)
Combined	3 (14.3)
Number of personnels teaching DPH: median (min, max)	3 (1, 25)
Who teaches DPH ^¶^	
DPH academics	12 (57.1)
Pediatric Dentistry academics	15 (71.4)

^¶^ Percentages do not add up to 100 because multiple responses were allowed

Table 3 shows that DPH was taught in mainly the 3rd to 5th years/ program levels of the 5-year BDS dental programs using face to face lectures, seminars and projects. The most common methods of assessment were written exams including close ended questions such as multiple-choice questions and open-ended questions such as essays. The basis for DPH courses’ design was international guidelines and national accreditation standards.

**Table 3:** DPH teaching and assessment in the Egyptian dental schools participating in the survey

Factor	N (%)
Program level ^¶^	
1st year	0
2nd year	2 (9.5)
3rd year	10 (47.6)
4th year	5 (23.8)
5th year	10 (47.6)
Methods of teaching ^¶^	
Face to face lectures	21 (100)
Online lectures	7 (33.3)
Practical training sessions	5 (23.8)
Problem based learning	8 (38.1)
Seminars	20 (95.2)
Community outreach activities	5 (23.8)
Projects	12 (57.1)
Methods of assessment ^¶^	
Open-ended questions/ essays	15 (71.4)
Close ended questions/ MCQs	20 (95.2)
Assignments	16 (76.2)
Presentations	10 (47.6)
Case presentations	3 (14.3)
Requirements	7 (33.3)
Posters	8 (38.1)
Community service activities	9 (42.9)
Basis for course design ^¶^	
National development plan	8 (38.1)
Oral health needs of the community	9 (42.9)
National accreditation standards	12 (57.1)
International guidelines	15 (71.4)

^¶^ Percentages do not add up to 100 because multiple responses were allowed

**Fig. 1:**
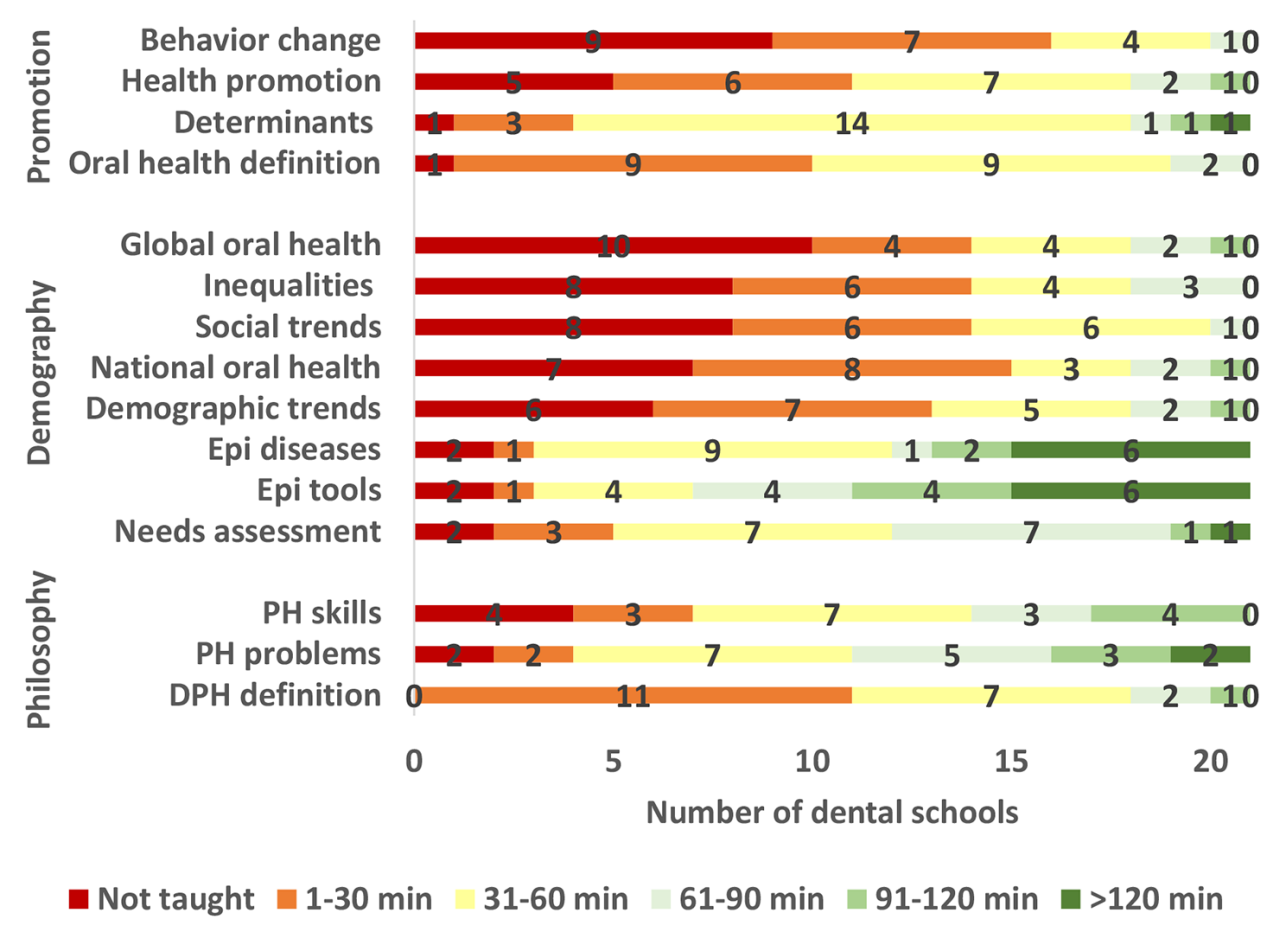
Time in minutes allocated by Egyptian dental schools to teaching DPH philosophy and approach, population demography and health and health promotion and disease prevention

**Fig. 2:**
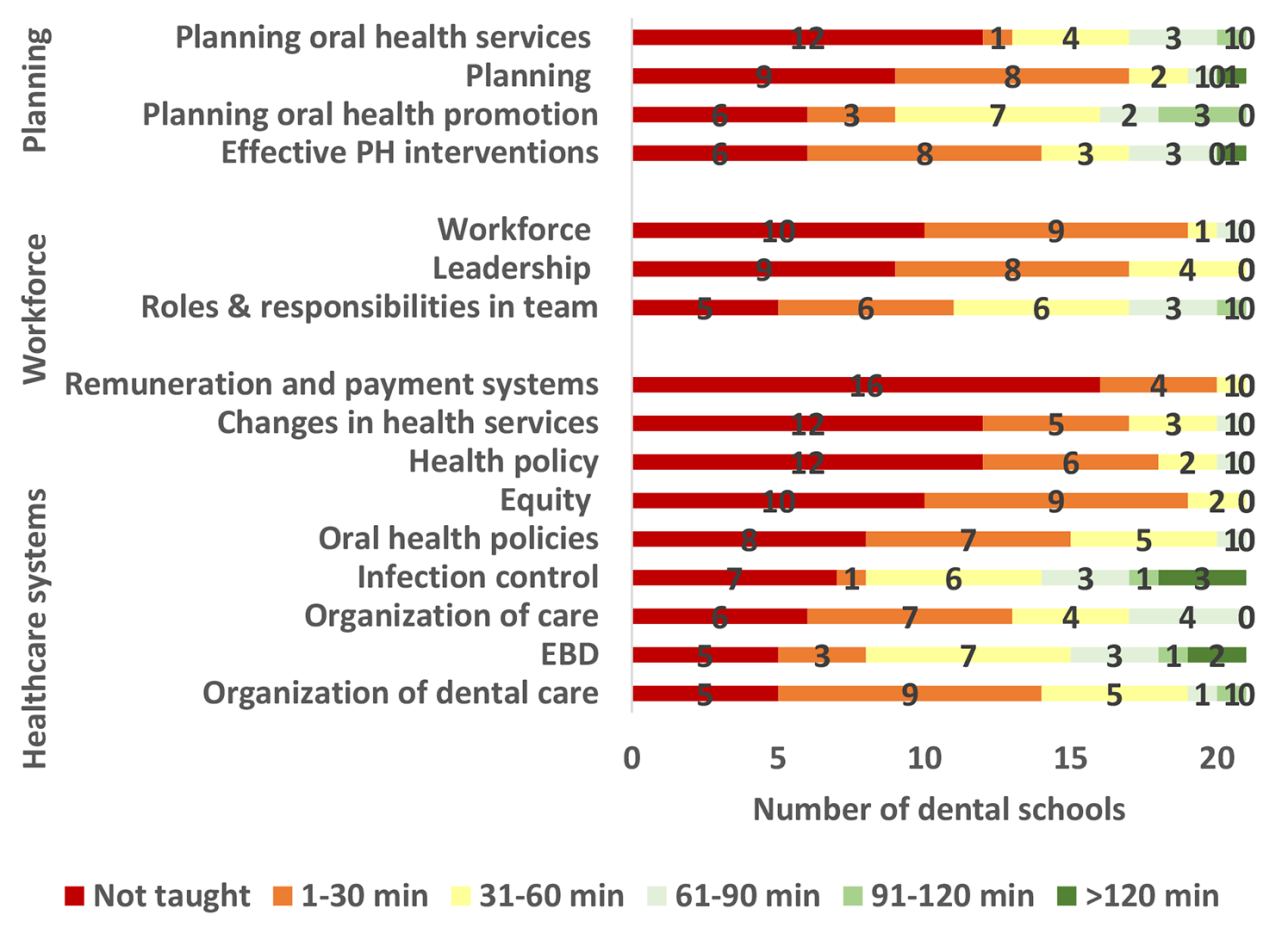
Time in minutes allocated by Egyptian dental schools to teaching healthcare systems, oral and dental workforce, and planning for health

**Fig. 3:**
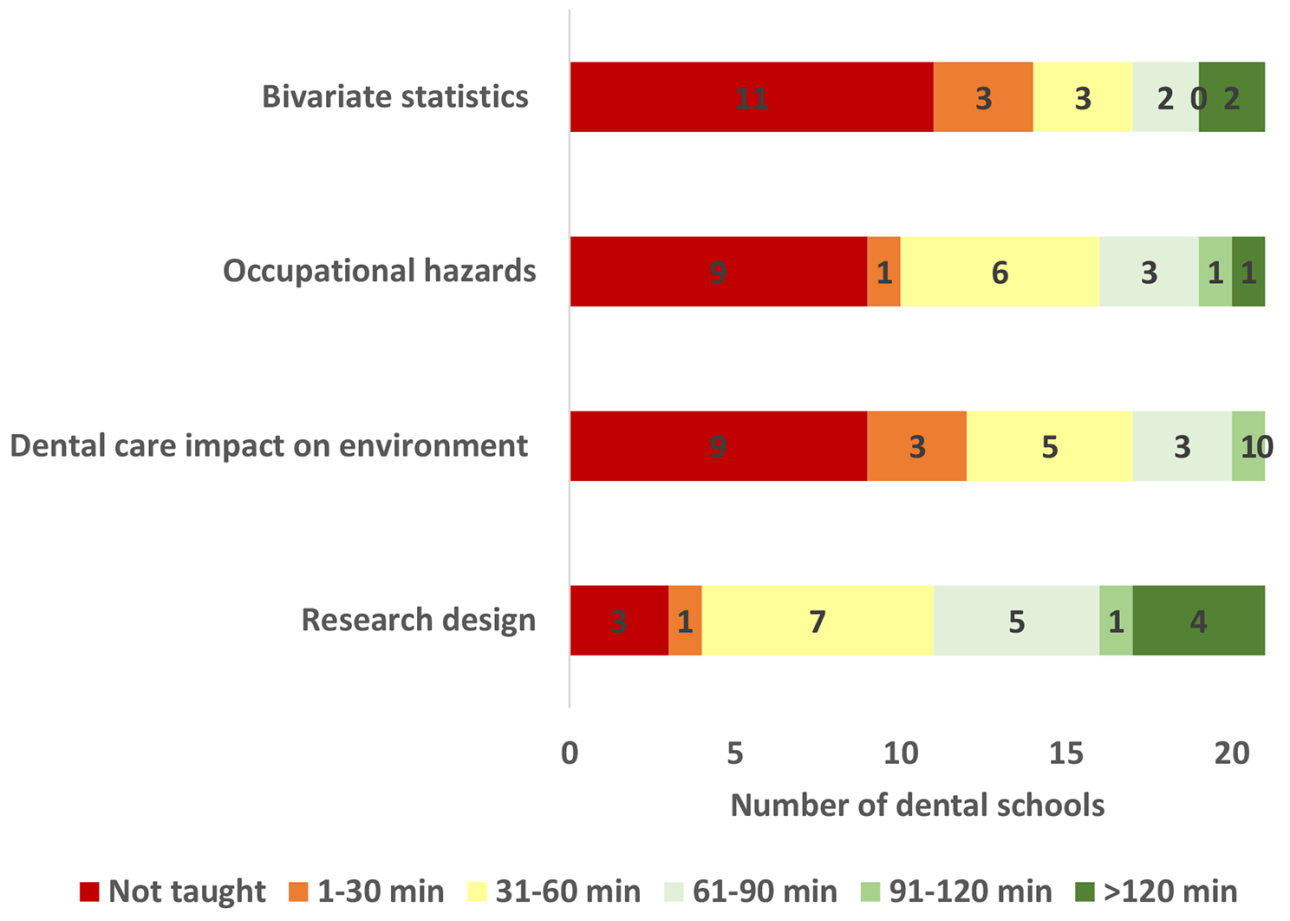
Time in minutes allocated by Egyptian dental schools to teaching additional topics in DPH

Figs. 1, 2 and 3 show variation among the participating Egyptian dental schools in teaching various DPH topics. DPH philosophy and approach as well as health promotion and disease prevention were covered by the greatest number of dental schools whereas oral and dental workforce and planning for health topics were covered by the least number of dental schools. All schools taught the definition of DPH and > 95% of schools taught the definition of oral health, the determinants of oral diseases and the prevention of oral diseases. Only five dental schools taught about remuneration and payment systems and four of them covered the subject in 30 min. The topic “Epidemiologic tools” was covered in the longest time (median = 91–120 min), followed by what constitutes public health problems, infection control, research design and prevention of oral diseases where each was covered in 61–90 min. Most topics in healthcare systems and dental/ oral workforce were covered in less than 30 min.

## Discussion

This is the first study to address DPH education in BDS programs in Egyptian dental schools. In the Egyptian dental schools, DPH education focused mostly on disease determinants, prevention, needs assessment, epidemiology of oral diseases and principles of research design. DPH was generally taught as lectures and seminars in senior years by a median of three academics who were pediatric dentistry academics in most cases with courses developed according to international guidelines. Minimal focus was given to teaching through community outreach activities. Students’ assessment was mainly based on written exams, and a minority of dental schools used case presentations. Healthcare systems, workforce issues and planning for health were not included in the DPH curricula in many dental schools, and if included, the least time was allocated for them.

The present study, based on the international model [9], has its strengths and limitations. The strength was that the survey and questionnaire topics were based on a comprehensive European DPH education survey [9]. However, there were limitations. First, the response rate was modest, partly due to the recent increase in the number of newly opened dental schools in the country. These new schools may not have reached the stage when DPH is taught. Thus, these schools may have declined to respond since they have no DPH education experience to report. When data from established dental schools are analyzed, the response rate becomes much higher. Second, another potential source of limitation is the degree to which the respondent was aware of how DPH was taught. There is a possibility of over or underestimating the time allocated to each topic because of this. However, we ensured that responses were collected only from academics with official posts who were authorized to report on the programs.

The study showed that teaching DPH in Egyptian dental schools focused on oral epidemiology, prevention, and research design. This differs from the emphasis laid in American schools [21] on social and healthcare systems, cultural competency, and oral health promotion. Our findings also disagree with the recommendations of the American Academy of Public Health Dentistry [22] which focus on teaching health disparities, surveillance, global oral health, racism in the healthcare system, the impact of poverty on oral health, the role of tobacco and nutrition in oral health as well as the integration of evidence-based dentistry into multiple DPH topics. By contrast, European dental schools [9] focus on population health, health promotion, planning of health promotion measures while the Nordic dental schools [23] emphasize the teaching of healthcare systems and global oral health development. These differences in topics reflect the national healthcare systems, political structures, priorities, and approaches. For example, cultural competency is relevant to the multi-ethnic American society where subpopulation groups from different cultures seek care and healthcare professionals need to be able to interact with them appropriately [24]. This competency may be less relevant in countries with different population profiles such as in Egypt. Also, preparing graduates to conduct surveillance activities will not be practical if the healthcare system at the country level, as in Egypt, does not have an oral health surveillance system.

The study showed that the percentage of pediatric dentistry academics teaching DPH was greater than DPH academics. This may be partly explained by the development of dental postgraduate degrees in Egyptian universities. Several universities award a combined master’s degree in Pediatric and Preventive Dentistry while only a few universities award a PhD in DPH. Thus, the opportunities for specialization in DPH are limited resulting in reduced availability of DPH specialists [25].

The present study showed that teaching DPH in Egyptian dental schools was traditional, and mainly lecture-based. This method, although cost effective, has limited impact on knowledge acquisition [26] and minimal potential for developing practical or cognitive skills. By contrast, evidence-based [27] and problem-based [28] methods of teaching promote critical thinking skills and lifelong and independent learning in students. More immersive methods based on real life experiences are available, too. For example, a study from Peru [29] showed that a competency-based curriculum was utilized, in which students train in low-income urban and rural communities. This exposed the students to challenges that were present in the community, thus building their capacities at an early stage of their careers. Community-based training increases the chances of developing solutions to problems that have better fit to the community needs and resources as opposed to learning through lectures that rely on educational resources generally produced in higher income countries with different oral health challenges and care systems [30]. Another advantage of community-based learning is utilizing the Ministry of Health public clinics where students can train on delivering primary healthcare services, thus reducing the need for campus-based training facilities, which is a great advantage considering the large class size observed in this study.

The study also showed that traditional student assessment methods, such as written examinations, were mostly used in Egyptian dental schools. Alternative methods such as community outreach activities help students apply the skills they learn [31] and contribute to covering the community needs. This method of assessment fits with community-based learning, aligns with the concepts of DPH and builds links for multi sectoral partnership between stakeholders in higher education, health, and social services as well as non-governmental organizations, thus developing students’ advocacy and community involvement skills. In a middle-income country such as Egypt, these skills are important for healthcare professionals including dentists [32]. It is also important to prepare dental educators for these methods of teaching and assessment by first training the educators since these skills are usually not part of the specialty training that they receive.

DPH curriculum in most Egyptian dental schools was based on international guidelines for DPH education. These guidelines need to be updated in view of the recent developments in the field of oral health at a global level. The World Health Assembly [33] emphasized the need to merge oral healthcare with the noncommunicable diseases (NCDs) agenda and transition from curative to preventive care under the umbrella of universal health coverage (UHC). The WHO also recently included dental products such as fluoride, glass ionomer cement, and silver diamine fluoride in the List of Essential Medicines for Children [34] and, together with the International Dental Federation, is pushing for amalgam phase-down [35]. Provision of oral healthcare for digital natives is another priority with increasing importance, especially post the COVID-19 pandemic. Even at a national level, new developments in health conditions and health policies call for a revision of healthcare workforce training strategies. Egypt is progressing towards UHC and implementing the Social Health Insurance Act to increase citizens’ access to healthcare services without financial hardships [36]. Also, the WHO statistics [36] show that 84% of the mortality in Egypt is related to NCDs which are interlinked with oral health. National priorities should include the control of risk factors shared between oral diseases and NCDs such as tobacco, which is used by 22% of adult Egyptians [37]. These developments clearly show that traditional oral healthcare models need to be replaced by new strategies with changes reflected in educational curricula. A new philosophy is needed to guide the restructuring of DPH education in Egyptian dental schools.

This study presents a comprehensive overview about DPH undergraduate education in Egyptian dental schools. It shows the need for information regarding DPH education in postgraduate studies. Also, the scarcity of data about curricula to train healthcare workers in DPH outside western countries calls for more studies in the Middle East and Africa. The need for the modernization and standardization of DPH teaching is clear especially with increasing mobilization of healthcare providers nowadays [7]. Educators, practitioners and policy makers need to establish a set of DPH skills and competencies to be developed in BDS programs in Egypt in addition to ensuring that DPH education is a core component of BDS programs.

### Electronic supplementary material

Below is the link to the electronic supplementary material.

Supplementary Material 1

## Data Availability

The data used and analyzed in the present study are available from the corresponding author on reasonable request.

## Abbreviations

DPH: Dental Public Health
WHO: World Health Organization
BDS: Bachelor of Dental Surgery
UHC: Universal health coverage
