# Supplementary material for: Dental public health education in Egypt: a cross-sectional survey
Source: BMC Med Educ. 2023 Nov 25;23:899. doi: 10.1186/s12909-023-04888-9 (PMC10675885; doi:10.1186/s12909-023-04888-9)
Supplement: Supplementary file 1 — Supplementary Material 1 [file 12909_2023_4888_MOESM1_ESM.docx]

**Appendix 1**

This survey assesses the current practices of teaching Dental Public Health (DPH) to undergraduate dental students in Egypt. All questions are about the Bachelor degree only. Please provide responses that describes the actual situation in your institution to the best of your ability.

1. **PROFILE OF THE FACULTY OF DENTISTRY**
2. What is the name of your University? _______________
3. Which type of university is your university?

- Public
- Private
- National
- Affiliated with religious bodies (Al-Azhar)
- Others

1. On average, how many dental students currently graduate per year from the Faculty of Dentistry you are affiliated with?

Number of students: _________

1. Please check all that describes the Faculty of Dentistry where you work:

- Teaching institution
- Research institution
- Provides undergraduate program
- Provides postgraduate program

1. **TEACHING AND ASSESSMENT OF DENTAL PUBLIC HEALTH**
2. Is there a dedicated Department or subdivision of a Department that teaches Dental Public Heath (DPH) in your Faculty?

- Yes, there is a whole Department that only teaches DPH
- Yes, there is a subdivision of a Department that only teaches DPH
- No, there is no specific Department or subdivision of a department teaching DPH

1. If you answered yes to the previous question, what is the name of the Department or subdivision of a Department?

- Department of Dental Public Health
- Department of Community Dentistry
- Department of Preventive Dentistry
- Subdivision of Dental Public Health in a Department
- Subdivision of Community Dentistry in a Department
- Subdivision of Preventive Dentistry in a Department
- Other (*Please specify*) ____________________

1. If you answered “No”, which Department/s co-ordinate undergraduate teaching in DPH/ Community Dentistry. *Please tick all that apply*.

- Paediatric Dentistry
- Conservative/ Restorative Dentistry
- Oral Medicine and Periodontology
- No specific teaching in DPH
- Other (*Please specify*) ____________________

1. How many persons teach/ coordinate the teaching of DPH in your Faculty?

_________________

1. In which year/s of undergraduate education is DPH/ Community Dentistry taught? *Please tick all that apply*.

- Year 1
- Year 2
- Year 3
- Year 4
- Year 5
- Year 6
- All Years

1. Who teaches the DPH/ Community Dentistry elements of the undergraduate programme? *Please tick all that apply*.

- Academics in Dental Public Health
- Academics in Public Health
- Academics in Paediatric Dentistry
- Academics in Restorative Dentistry
- Other (*Please specify*) ____________________

1. How is DPH/ Community Dentistry taught at your school? *Please tick all that apply*:

- Lectures
- Seminars
- Workshops
- Online Tools
- Outreach visits
- Problem Based Learning
- Project Work
- Other (*Please specify)* ____________________

1. How is DPH/ Community Dental Health assessed at your Faculty? *Please tick all that apply*:

- Examinations: Essay questions
- Examinations: Multiple choice questions
- Coursework/ assignments
- Other (*Please specify*) ____________________

1. **COURSE CONTENT**
2. **Which of these describe the basis for designing the DPH content in the undergraduate program?** *Please tick all that apply*:

- **It follows the national developmental plan for Egypt.**
- **It addresses the oral health needs in Egypt.**
- **It follows he standards of accreditation in Egypt.**
- **It is based on international guidelines for teaching DPH in undergraduate dental programs.**

1. **The next set of questions asks about the topics currently taught in your Faculty of Dentistry**

|  | 0 | 0-30 min | 31-60 min | 61-90 min | 91-120 min | > 120 min |
| --- | --- | --- | --- | --- | --- | --- |
| 1. Definitions of dental public health |  |  |  |  |  |  |
| 1. Public health approach and skills |  |  |  |  |  |  |
| 1. Consideration of diseases which are public health problems |  |  |  |  |  |  |
| 1. Oral health needs assessment |  |  |  |  |  |  |
| 1. Epidemiological tools (e.g. indices) and indicators |  |  |  |  |  |  |
| 1. Epidemiology of oral disease |  |  |  |  |  |  |
| 1. Demographic trends |  |  |  |  |  |  |
| 1. Social trends |  |  |  |  |  |  |
| 1. National oral health trends in Egypt |  |  |  |  |  |  |
| 1. Global oral health trends |  |  |  |  |  |  |
| 1. Inequalities in oral health |  |  |  |  |  |  |
| 1. Concepts and definition of oral health |  |  |  |  |  |  |
| 1. Determinants of oral health |  |  |  |  |  |  |
| 1. Evidence base for oral health promotion |  |  |  |  |  |  |
| 1. Behaviour change |  |  |  |  |  |  |
| 1. Health policy |  |  |  |  |  |  |
| 1. Organisation and delivery of healthcare in general |  |  |  |  |  |  |
| 1. Organisation and delivery of public & private dental care |  |  |  |  |  |  |
| 1. Equity of care |  |  |  |  |  |  |
| 1. Infection control |  |  |  |  |  |  |
| 1. Evidence based health care |  |  |  |  |  |  |
| 1. Remuneration and payment systems |  |  |  |  |  |  |
| 1. Examples of change in health services |  |  |  |  |  |  |
| 1. Oral health policies |  |  |  |  |  |  |
| 1. Roles and responsibilities within dental team |  |  |  |  |  |  |
| 1. Dental leadership |  |  |  |  |  |  |
| 1. The dental public health workforce |  |  |  |  |  |  |
| 1. Theory of planning |  |  |  |  |  |  |
| 1. Examples of effective public health interventions |  |  |  |  |  |  |
| 1. Planning oral health promotion |  |  |  |  |  |  |
| 1. Planning oral and dental services |  |  |  |  |  |  |
| 1. Research design and types of epidemiological studies |  |  |  |  |  |  |
| 1. Bivariate statistical analysis |  |  |  |  |  |  |
| 1. Impact of dental care on the environment |  |  |  |  |  |  |
| 1. Occupational hazards in dentistry |  |  |  |  |  |  |

1. Please add any final comments on this subject or the questions.
2. Thank you for completing this questionnaire. Please provide your title/ role:

- Dean
- Vice Dean for Education and Students’ Affairs
- Head of Department
- Professor of Dental Public Health/ Community Dentistry
- Other person within the school who is knowledgeable about the subject of Dental Public Health
- Other (*Please specify*) ____________________

**Appendix 2**

**List of Egyptian dental schools by participation status and date of foundation**

| University | Responded Yes/ No | Year of foundation | Postal address Yes/ No | E-mail sent Yes/ No |
| --- | --- | --- | --- | --- |
| AAST - Alamein | Yes | 2018 | Yes | No email |
| Ahram Canadian University | Yes | 2013 | Yes | Yes |
| Ain Shams University | Yes | 1994 | Yes | Yes |
| Alamein International University | No | 2020 | Yes | No email |
| Alexandria University | Yes | 1945 | No | Yes |
| Alsalam University | No | 2018 | Yes | Yes |
| Assiut University | Yes | 2012 | Yes | Yes |
| Azhar (Assiut) University | Yes | Not Available | No | Yes |
| Azhar University - Boys | Yes | Not Available | No | WhatsApp, no email |
| Azhar University - Girls | Yes | Not Available | No | WhatsApp, no email |
| Badr University | No | 2014 | Yes | Yes |
| Beni Suef University | No | 2013 | Yes | Yes |
| British University in Egypt (BUE) | Yes | 2012 | Yes | Yes |
| Cairo University | Yes | 1925 | Yes | Yes |
| Delta University | Yes | 2011 | Yes | Yes |
| Deraya University | No | 2020 | Yes | Email not available |
| Egyptian Russian University (ERU) | Yes | 2006 | Yes | Yes |
| Fayoum University | Yes | 2012 | Yes | Yes |
| Future University in Egypt (FUE) | No | 2006 | Yes | Yes |
| Galala University | No | 2020 | No | No email |
| Horus University | No | 2016 | Yes | Yes |
| Kafrelsheikh University | Yes | 2014 | Yes | Yes |
| King Salman International University | No | 2020 | Yes | Yes |
| Mansoura University | No | 1977 | Yes | Yes |
| Menoufia University | No | 2021 | Yes | Yes |
| Minia University | No | 1995 | Yes | Yes |
| Misr International University (MIU) | Yes | 1996 | Yes | Yes |
| Misr University for Science and Technology (MUST) | Yes | 1996 | Yes | Yes |
| Modern University for Technology and Information (MTI) | No | 2009 | Yes | Undeliverable |
| MSA University | Yes | 2006 | No | WhatsApp, no email |
| Nahda University in Beni Suef | Yes | 2014 | Yes | Yes |
| New Mansoura University | No | 2021 | Yes | No email |
| New Giza University | No | 2016 | Yes | Yes |
| October 6 University | No | 1998 | Yes | Yes |
| Pharos University | Yes | 2006 | Yes | Yes |
| Sinai University – Arish Campus | No | 2006 | No | Yes |
| Sinai University – Qantara Campus | No | 2005 | No | Undeliverable |
| South Valley University | No | 2013 | Yes | Yes |
| Sphinx University | Yes | 2019 | Yes | Yes |
| Suez Canal University | No | 1996 | Yes | Yes |
| Suez University | No | 2020 | Yes | Yes |
| Tanta University | Yes | 1973 | Yes | Yes |
| Zagazig University | No | 2016 | Yes | Yes |
